# Outcome of adult patients with JIA treated with the biosimilar Benepali^®^: results of the biologic register JuMBO

**DOI:** 10.1186/s13075-022-02968-7

**Published:** 2022-12-13

**Authors:** Kristina Vollbach, Klaus Tenbrock, Nobert Wagner, Gerd Horneff, Ariane Klein, Ivan Foeldvari, Johannes-Peter Haas, Peer Aries, Georg Gauler, Frank Striesow, Paula Hoff, Christine Scholz, Stefanie Tatsis, Eva Seipelt, Jens Klotsche, Kirsten Minden

**Affiliations:** 1 Department of Pediatrics, RWTH Aachen University Hospital, Aachen, Germany; 2 Centre for Pediatric Rheumatology, Department of Pediatrics, Asklepios Clinic Sankt Augustin, Sankt Augustin, Germany; 3 Department of Pediatric and Adolescent Medicine, University Hospital of Cologne, Cologne, Germany; 4 Zentrum für Kinder- und Jugendrheumatologie, Hamburg, Germany; 5 German Centre for Pediatric and Adolescent Rheumatology, Garmisch-Partenkirchen, Germany; 6 Rheumatologie im Struenseehaus, Hamburg, Germany; 7 Rheumapraxis an der Hase, Osnabrück, Germany; 8 Internistisch-Rheumatologische Praxis, Bonn, Germany; 9 MVZ Endokrinologikum Berlin am Gendarmenmarkt, Berlin, Germany; 10 Internistisch-Rheumatologische Praxis, Freiburg, Germany; 11 Marienkrankenhaus, Hamburg, Germany; 12 Immanuel Krankenhaus, Berlin Buch, Germany; 13 Deutsches Rheuma-Forschungszentrum Berlin, Leibniz Association, Berlin, Germany; 14 Charité - Universitätsmedizin Berlin, corporate member of Freie Universität Berlin and Humboldt-Universität zu Berlin, Berlin, Germany

**Keywords:** Juvenile idiopathic arthritis, Biosimilar, Etanercept, Enbrel^®^, Benepali^®^, Effectiveness, Therapy survival

## Abstract

**Background:**

To analyze therapy adherence, safety, and outcome in adult patients with juvenile idiopathic arthritis (JIA) treated with the etanercept biosimilar Benepali^®^ (Biogen Inc, Cambridge, USA).

**Methods:**

Data from the prospective registry, JuMBO (Juvenile arthritis MTX/Biologics long-term Observation), were used for the analysis. JuMBO is a long-term observational cohort study. It follows adult patients with JIA who were formerly included in the national JIA biologic register (BiKeR Registry). Both registries provide individual trajectories of clinical data and outcomes from childhood to adulthood in JIA patients treated with disease-modifying anti-rheumatic drugs (DMARDs).

**Results:**

Eighty-three patients from the German JuMBO registry were treated with Benepali^®^. Of these, 74% had switched from Enbrel^®^ (Pfizer Inc., NYC, USA) the originator of etanercept to Benepali^®^ for cost reasons. Therapy survival of patients treated with Benepali^®^ in comparison to Enbrel^®^ in patients matched by significant parameters was comparable. Adverse events (AE) were reported in 25.3% and serious adverse events (SAE) in 9.6% of patients. Physicians rated no SAE causative related to Benepali^®^. The majority of SAEs were surgical/medical procedures and there was only one infection. All efficacy parameters (cJADAS-10, Physician Global Assessment, number of joints with active arthritis, patients’ overall well-being, pain, and HAQ) demonstrated improvement over 24 months (*p*-values were not significant). 9.6% of patients permanently discontinued Benepali® because of an AE.

**Conclusions:**

Tolerability and effectiveness of the biosimilar Benepali^®^ were satisfactory and therapy survival was comparable to the originator. Further data on therapy with biologics and biosimilars such as Benepali^®^ must be collected by registries such as BiKeR and JuMBO in order to optimize therapy and patient outcomes and to reduce costs in the health system in the long term.

## Background

Juvenile idiopathic arthritis is the most common chronic rheumatic disease of childhood and a leading cause of short- and long-term disability [1]. The use of biologics has dramatically changed the therapeutic approach and associated outcomes. Since the approval of the first biologic, the BiKeR and JuMBO registers have provided data on long-term effectiveness and safety for therapy with DMARDs (disease-modifying anti-rheumatic drugs) in JIA [2–5].

Enbrel® (Pfizer Inc., NYC, USA) is still the biological agent most frequently used in Germany for JIA to achieve inactive disease or remission and to prevent long-term consequences [6].

The introduction of biosimilar DMARDs (bsDMARDs) has the potential to improve patient access to biologic therapy and to improve overall patient outcomes while they can lower the costs in health care [7]. This is why the German Joint Government Committee of physicians and health insurance companies has adopted guidelines for an economical way of prescribing biologics and biosimilars, which in the opinion of the Committee could lead up to savings of 37% compared with the use of original preparations [8]. Currently, there are three biosimilars approved for the etanercept originator Enbrel^®^ in the EU: Benepali^®^ (Biogen Inc, Cambridge, USA), Erelzi^®^ (Novartis AG, Basel, Switzerland), and Nepexto^®^ (Mylan Inc., Canonsburg, USA).

Since the therapy with a DMARD often has to be continued over a long period of time and may be crucial for the clinical outcome, data concerning the long-term safety and effectiveness of these substances are highly relevant. Data on the safety and effectiveness of bsDMARDs in JIA are still very sparse; there are still theoretical concerns about biosimilars regarding reduced efficacy, altered immunogenicity, and a different safety profile compared to the biooriginator [9].

Here we analyzed data concerning therapy adherence, safety, and clinical outcome for 83 patients from the JuMBO registry being treated with Benepali^®^ to determine whether therapy is sufficient and safe.

## Methods

### Data sources

Data from JIA patients who reached 18 years of age were transferred from the JIA registry Biologics in Pediatric Rheumatology (BiKeR) [3] to its follow-up (FU) registry, Juvenile arthritis Methotrexate/Biologics long-term Observation (JuMBO) [10]. Both are ongoing multicenter prospective observational cohort studies. JuMBO was approved by the Ethics Committee of the Charité University Medicine Berlin and conducted in accordance with the Declaration of Helsinki.

Both registers aim to monitor the safety and effectiveness of cs and/or bDMARDs in patients with JIA [6, 11–13]. JuMBO monitors patients who have reached the age of 18 or left pediatric rheumatology care. Previous reports have extensively described the design of both registers [2, 10, 14, 15].

### Patients

All patients with JIA according to the International League of Associations for Rheumatology criteria [16] who were enrolled in BiKeR in childhood, subsequently transferred to JuMBO, and ever treated with etanercept and the etanercept biosimilar Benepali^®^ were considered for this study. Written informed consent was obtained from the patients (age ≥18 years).

### Procedures and assessments

In JuMBO, patients are assessed every 6 months. The patient questionnaire includes socio-demographic characteristics, educational background, periods of incapacity for work, and hospitalizations. Patients rate their general well-being, disease activity, pain, and fatigue on an 11-point numerical rating scale (NRS, 0-10). In addition, the patient-relevant outcome instrument Health Assessment Questionnaire (HAQ) is used [17].

The doctor's questionnaire collects information on the current clinical condition of the patient (e.g., number of joints with swelling, range of motion limitations, tenderness or pain with motion (72-joint count)), erythrocyte sedimentation rate and C-reactive protein levels, and a physician’s global assessment of the patient’s disease activity (PhGA) on NRS. Current therapies, reasons for discontinuing them, and (serious) adverse events (AEs/SAEs) are documented as well.

Disease activity was assessed by the clinical Juvenile Arthritis Disease Activity Score (cJADAS-10) [18]. The cJADAS-10 cut-off values for defining the different states of disease activity were based on definitions by Consolaro et al. [19].

An AE is defined as any untoward medical occurrence associated with a pharmaceutical product during observation; a serious AE (SAE) results either in death, life-threatening illness, hospitalization/prolongation of hospitalization or medical or surgical intervention to prevent a serious outcome, results in persistent/significant disability or incapacity, or is a congenital anomaly or birth defect or a medically significant event determined by the responsible local physician that in their opinion jeopardized the health of the patient and required intervention to prevent one of the other outcomes listed [20]. AEs and SAEs were coded on the preferred term level with MedDRA (Medical Dictionary for Regulatory Activities) version 23.0.

### Statistical analysis

Descriptive statistics included frequencies for categorical data and means, standard deviations, and median values for continuously distributed data. Therapy survival was investigated by Kaplan-Meier method. A matched etanercept originator (Enbrel^®^) control group was used in order to compare therapy survival with Benepali^®^. An Enbrel^®^-treated patient was matched to a Benepali^®^ patient by number of bDMARD treatment courses in the past, JIA category, sex, ANA positivity, and disease duration at treatment start (± 3 years). The course of clinical parameters after treatment start with Benepali^®^ was investigated in patients who switched from the etanercept originator to Benepali^®^ for cost reasons by generalized linear mixed models. Time of follow-up was estimated with a linear spline approximation in order to model the heterogeneously distributed survey times in follow-up. All assessments within 3 months before start of Benepali^®^ therapy and up to 3 months after the end of Benepali^®^ therapy were used for the analyses. All models were adjusted for treatment duration with the etanercept originator before the switch to Benepali^®^. AE and SAE were reported by the number of patients with at least one such an event, number of events, and with crude incidence rates (IR) per 100 exposure years to Benepali^®^. 95% confidence intervals for incidence rates were calculated by Poisson rate confidence intervals for all AE and SAE. A *p*<0.05 was considered statistically significant. Data were analyzed using SAS V.9.4 software.

## Results

From a total of 1844 patients included in the German JuMBO registry, 83 patients were identified on June 23, 2021, who have ever been treated with Benepali^®^.

### Patient characteristics

The patient characteristics are shown in Table 1. Most patients started Benepali^®^ treatment after the age of 18 with a range of 16.6 to 38.2 years.

**Table 1:** Patient characteristics

	Total cohort	Matched cohort	
	Benepali^®^	Benepali^®^	Enbrel^®^
	***n***=83	***n***=71	
Female, *n* (%)	60 (72.3%)	51 (71.8%)	52 (73.2%)
Age at JIA onset, mean (SD), median	8.8 (4.7), 9.4	8.7 (4.6), 9.8	8.7 (4.6), 9.8
sJIA, *n* (%)	3 (3.6%)	3 (4.2%)	3 (4.2%)
RF negative PA, *n* (%)	22 (26.5%)	19 (26.7%)	19 (26.7%)
RF positive PA, *n* (%)	9 (10.8%)	7 (9.9%)	7 (9.9%)
Enthesitis-related arthritis, *n* (%)	17 (20.5%)	16 (22.5%)	15 (21.1%)
Psoriatic arthritis, *n* (%)	4 (4.8%)	4 (5.6%)	4 (5.6%)
Other arthritis, *n* (%)	5 (6.0%)	5 (7.0%)	5 (7.0%)
Oligoarthritis, persistent, *n* (%)	4 (4.8%)	2 (2.8%)	2 (2.8%)
Oligoarthritis, extended, *n* (%)	19 (22.9%)	15 (21.1%)	16 (22.5%)
ANA positive, *n* (%)	31 (37.8%)	26 (36.6%)	27 (28.0%)
HLA B27 positive, *n* (%)	27 (32.9%)	24 (33.8%)	23 (32.4%)
Uveitis/history, *n* (%)	2 (3.8%)	1 (2.1%)	1 (2.1%)
Disease duration at start of treatment with Benepali^®^, years, mean (SD), median	13.8 (6.2), 13.7	13.6 (5.9), 13.1	6.1 (7.4), 7.1
Age at start of treatment with Benepali^®^, mean (SD), median	22.5 (4.4), 21.3	22.1 (4.3), 21.2	14.7 (6.1), 15.3

### Previous therapy

For only 6 (7.2%) patients Benepali^®^ was the first-line biologic DMARD, the largest subgroup (*n*=31, 37.4%) had been treated with one bDMARD before switching to Benepali^®^. Over the course of the disease, the patients had received an average of 2.1 (SD 1.7, range 0 to 10) treatment courses with different biologic DMARDs prior to Benepali^®^. The last bDMARDs before starting Benepali^®^ was Enbrel^®^ (78.3%), adalimumab (3.6%), certolizumab (3.6%), baricitinib, Erelz﻿i^®^, tocilizumab, ustekinumab, and tofacitinib (1.2% each).

Fifty-seven of 77 patients (74.0%) discontinued their previous bDMARD to switch from Enbrel^®^ to Benepali^®^ for cost reasons. Other reasons for discontinuation of prior bDMARD therapy included inactive disease (11.7%) and ineffectiveness (14.3%). Adverse events led to the termination of therapy in 5.2% and in 3.9% other reasons were causative.

### Therapy survival

At the time of analysis, 54 of 83 (65.1%) patients were still on therapy while 29 patients had terminated therapy with Benepali^®^. The reasons for discontinuing therapy were diverse and included, e.g., ineffectiveness, the occurrence of an adverse event, and inactive disease in decreased order (Table 2).

**Table 2:** Reasons for termination of therapy with Benepali^®^ (multiple answers were possible)

	***n***=83	Median survival time (95% CI) in years
**Ongoing therapy,** ***n*** **(%)**	54 (65.1%)	
**Therapy terminated,** ***n*** **(%)**	29 (34.9%)	3.6 (1.8–3.6)
**Reason for termination**		
Adverse event, n (%)	8 (9.6%)	
Inactive disease, n (%)	4 (4.8%)	
Ineffectiveness, n (%)	10 (12.1%)	
Other^a^, *n* (%)	7 (8.4%)	

^a^Pregnancy (2), patient request (1), Crohn’s disease (1), desire to conceive a child (1), subjective pain during injection (1), unknown (1)

**Fig. 1:**
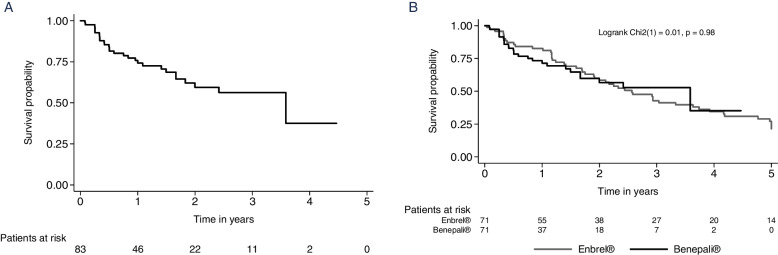
**A** Therapy survival of patients treated with Benepali^®^ (*n*=83) and **B** therapy survival of Benepali^®^ in comparison to Enbrel^®^ in patients matched by the number of bDMARD treatment courses in the past, JIA category, sex, ANA positivity, and disease duration at treatment start (± 3 years)

The Kaplan-Meier curve (Fig. 1A, *n*=83) for therapy adherence to Benepali^®^ shows that after a follow-up period of 2 years about 60% of the patients are still under therapy, while after 4 years about 30% are still under therapy with Benepali^®^. In total, for 71 (of 83) Benepali^®^-treated patients, Enbrel^®^-treated patients could be identified and matched. Benepali^®^ showed a comparable therapy survival as observed for Enbrel^®^ (Fig. 1B).

### Safety

For 8 (9.6%) patients, the reason to terminate therapy with Benepali^®^ was the occurrence of an adverse event. Upon closer inspection, any adverse event occurred in 21 patients (25.3%) while any serious adverse event occurred in 8 patients (9.6%). During the observation period of patients on Benepali^®^ comprising 145 exposure years, 40 AEs and 10 SAEs were reported, resulting in an AE rate of 27.6 (95% CI, 19.7–37.6) and an SAE rate of 6.9 (95% CI, 3.3–12.7) per 100 patient-years. No SAE was rated by physicians to be causally related to Benepali^®^.

The majority of SAEs were surgical/medical procedures (Table 3). Interestingly, among the SAEs was only one infection. Under the reported AEs, infections were the largest subgroup with a rate of 8.28 per 100 patient-years (95% CI, 4.28–14.46).

**Table 3:** Serious adverse events under therapy with Benepali^®^

System Organ Class term	Preferred Term (PT)	***n***=83	Number of SAE	145 exposure years
		***n*** (%)		AE rate per 100 exposure years (95% CI)
**Infections and infestations**		1 (1.2%)	1	0.69 (0.02–3.84)
	Urospesis	1 (1.2%)	1	0.69 (0.02–3.84)
**Pregnancy, puerperium and perinatal conditions**		2 (2.4%)	2	1.38 (0.17–4.98)
	Hyperemesis gravidarum	1 (1.2%)	1	0.69 (0.02–3.84)
	Premature birth	1 (1.2%)	1	0.69 (0.02–3.84)
**Respiratory, thoracic, and mediastinal disorders**		1 (1.2%)	1	0.69 (0.02–3.84)
	Asthma	1 (1.2%)	1	0.69 (0.02–3.84)
**Surgical and medical procedures**		5 (6.0%)	6	4.14 (1.52–9.01)
	Ankle operation	1 (1.3%)	1	0.69 (0.02–3.84)
	Parathyroidectomy	1 (1.3%)	1	0.69 (0.02–3.84)
	Renal stone removal	1 (1.3%)	1	0.69 (0.02–3.84)
	Sinus operation	1 (1.3%)	1	0.69 (0.02–3.84)
	Synovectomy	1 (1.3%)	1	0.69 (0.02–3.84)
	Tooth extraction	1 (1.3%)	1	0.69 (0.02–3.84)

### Effectiveness

**Fig. 2:**
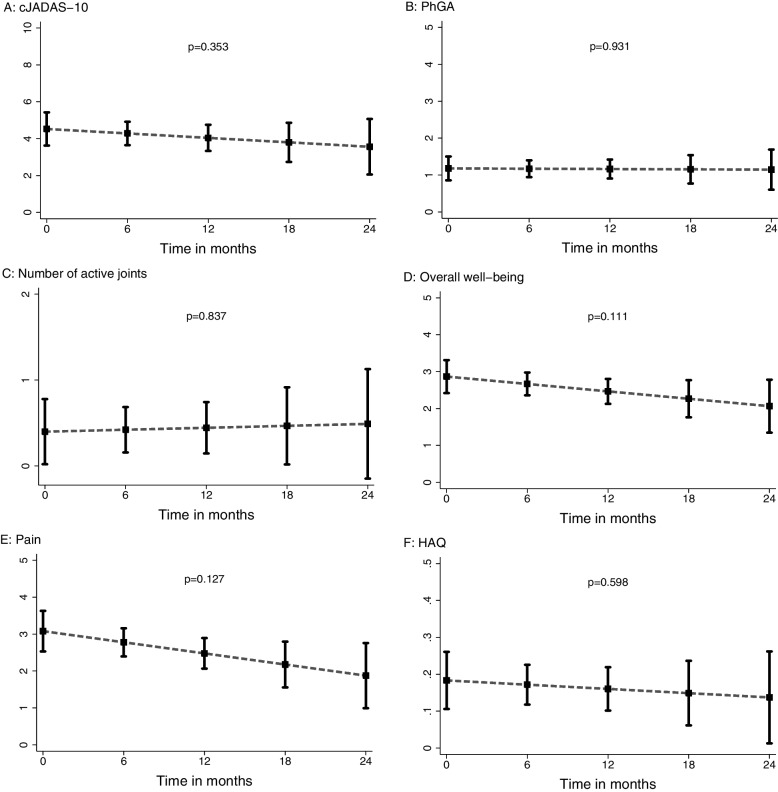
**A–F** **A** Course of cJADAS-10, **B** Physician Global Assessment (PhGA), **C** Number of joints with active arthritis, **D** Patients overall well-being (NRS 0-10), **E** Pain (NRS 0-19), and **F** HAQ values over time (predicted means with 95% confidence intervals estimated by generalized linear mixed models,  *p*-value for change in parameters over 24 months)

At the start of therapy with Benepali® in patients who switched from the etanercept originator to Benepali^®^ for cost reasons, the mean cJADAS10 was 4.5 (95% CI, 3.6–5.4), PhGA 1.2 (95% CI, 0.9–1.5), and the mean number of active joints 0.4 (95% CI, 0.1–0.8). On a numerical rating scale (NRS 0-10) patients stated their overall well-being at 2.9 (95% CI, 2.4–3.3) and their pain at 3.1 (95% CI, 2.5-3.6). HAQ-score at therapy start was 0.18 (0.11–0.26). At Benepali^®^ therapy start, 22.6% were in an inactive disease as assessed by cJADAS-10 (≤1), 85.3% had no active joint with arthritis, and 64.5% had no functional limitations (HAQ of 0). The course of the corresponding parameters in follow-up under Benepali^®^ therapy can be seen in Fig. 2A–F. Clinical JADAS-10, PhGA, overall well-being, pain, and HAQ were in decline, which indicates that Benepali® was successful in therapy. The *p*-values were not significant; however, since the major reason for switch of therapy was cost-effectiveness, this could contribute to the insignificant improvement of disease activity in addition to the still limited number of patients reported.

## Discussion

The JuMBO Registry provides long-term data of adult patients with juvenile idiopathic arthritis being treated with biologicals. To our knowledge, we herein present the first cohort of adult patients with onset of rheumatic disease in childhood treated with the biosimilar Benepali^®^.

The majority of the patients analyzed were female, which fits in with the general gender distribution of JIA. The distribution of the JIA categories showed a comparatively high proportion of patients with RF-negative polyarthritis and extended oligoarthritis which indicates a rather severe JIA spectrum with a higher likelihood of therapy failure. All patients included in this study had received Benepali^®^, and two-thirds of them were still on this drug at the time of this analysis. The therapy survival of Benepali^®^ was comparable to that observed for Enbrel^®^. This suggests a moderate side effect profile that can be tolerated by patients as well as a sufficient therapeutic effectiveness of Benepali^®^. This statement is also supported by the fact that ineffectiveness was the reason for discontinuing therapy in about 12% of the patients (especially considering the mentioned more severely affected patient cohort). In addition, a previous biological therapy before Benepali^®^ was terminated in 14.3% of the patients due to inefficiency.

An SAE was the reason for discontinuing therapy with Benepali^®^ for about 8% of patients. The rate of serious adverse events per 100 patient-years was about 1.2 times as high as the rate of patients on Enbrel^®^ in a cohort of 155 patients from the JuMBO Registry analyzed by Minden et al. in 2012 [10] and about 1.8 times as high as the rate of patients on etanercept in a group of 2725 patients analyzed by Armaroli et al. in 2020 [5]. In 2021, Thiele et al. described an increased rate of local reactions in their cohort of JIA patients treated with etanercept biosimilars during childhood compared with the originator which is not being reflected in our cohort reporting adult patients only [9]. On closer inspection, the listed SAEs are heterogeneously distributed and commonly reported SAEs (for example anaphylaxis, cytopenias, hepatic events) were not reported [15]. In addition, compared to other cohorts, no new autoimmune disease or uveitis was reported in the cohort examined [21]. However, the observation period was also comparatively short for these events. No SAE was rated to be causally related to Benepali^®^. The SAEs are more likely related to the age of the patient and the long disease duration of the patient cohort: 2 of the 10 SAEs were due to pregnancies and 2 to joint operations. Overall, the frequency and severity of the SAE are tolerable. This fits in with the findings of Thiele et al. who observed comparable safety profiles of biosimilars and the originator during childhood [9]. Since SAEs are rare events, the collection of more data in biologic registers is crucial to draw definite conclusions in the future.

An improvement from baseline was observed in all analyzed parameters (cJADAS10, Physician Global Assessment, number of joints with active arthritis, patient overall well-being, pain and HAQ) after a follow-up of 24 months. Because of the comparatively short treatment period and small number of patients, the results were not statistically significant but at least there was no sign of treatment failure. Cost aspects were the most common reason for switching the biologic therapy from Enbrel^®^ to Benepali^®^. Since almost one-quarter of patients had an inactive disease at the start of therapy with Benepali^®^, for these patients, little improvement of the well-controlled disease can be expected, so the observed preservation of this state is also a good outcome.

In adult patients, real-world evidence has so far provided an indication that Benepali^®^ is as effective and safe as the originator in both switched and naïve patients [22]. In Denmark, where there is a mandatory switch recommendation, Glintborg et al. found that the nationwide switch from originator to biosimilar etanercept in 1621 adult patients with inflammatory arthritis (rheumatoid arthritis, psoriatic arthritis, and axial spondyloarthritis) had no negative impact on 3 months’ disease activities, and no major safety events were observed [23]. Tweehuysen et al. reported a Dutch cohort of 635 adult patients with non-mandatory transitioning from the originator to Benepali^®^ which showed a slightly lower 6-month treatment persistence rate and smaller decreases in disease activity in the transition cohort compared to the historical cohort, but these differences were not considered clinically relevant [24].

In Germany, automatic substitution is currently not permitted for biologics and biosimilars. As mentioned above, in Denmark, there was a mandatory switch recommendation from the originator Enbrel^®^ to a biosimilar. Due to the introduction of new reference price group for TNF-α blockers including etanercept in Germany, the savings potential for the prescription of an etanercept biosimilar has decreased from about 6000€ to about 200 to 400€ per year and patient (for a weekly therapy with 50mg etanercept) compared to the originator Enbrel^®^ [25]. Glintborg et al. demonstrated that there were no obvious changes in overall use and costs of healthcare services following the switch from originator to biosimilar etanercept [26]. The question of these follow-up costs has not yet been addressed in German patients who are not mandatorily advised to switch.

In summary, a general similarity in efficacy and safety of Benepali^®^ compared to its originator Enbrel^®^ has been shown in the literature and is supported by our data. This will, however, have to be confirmed or refuted by the collection of further data in the future.

## Conclusion

Registers such as BiKeR and JuMBO provide valuable data on the safety and efficacy of therapy with DMARD in patients with JIA. To avoid long-term consequences of JIA, therapy with a bDMARD is often necessary. Knowledge of the effectiveness and safety of biologics and biosimilars such as Benepali^®^ is required in order to optimize therapy and patient outcomes and to reduce costs in the health care system in the long term.

## Data Availability

All data generated or analyzed during this study are included in this published article.

## Abbreviations

AE: Adverse event
ANA: Antinuclear antibodies
bDMARDs: Biologic DMARDs
BiKeR: Biologics in Pediatric Rheumatology Registry
bsDMARDs: Biosimilar DMARDs
CI: Confidence interval
cJADAS-10: Clinical Juvenile Arthritis Disease Activity Score
DMARDs: Disease-modifying anti-rheumatic drugs
HAQ: Health Assessment Questionnaire
IR: Incidence rate
JIA: Juvenile idiopathic arthritis
JuMBO: Juvenile arthritis MTX/Biologics long-term Observation
NRS: Numerical rating scale
PhGA: Physician Global Assessment
SAE: Serious adverse event
SD: Standard deviation
